# Observer Rated Sleepiness and Real Road Driving: An Explorative Study

**DOI:** 10.1371/journal.pone.0064782

**Published:** 2013-05-28

**Authors:** Anna Anund, Carina Fors, David Hallvig, Torbjörn Åkerstedt, Göran Kecklund

**Affiliations:** 1 Swedish Road and Transport Research Institute, Linköping, Sweden; 2 Stress Research Institute, Stockholm, Sweden; Bremen Institute of Preventive Research and Social Medicine, Germany

## Abstract

The aim of the present study was to explore if observer rated sleepiness (ORS) is a feasible method for quantification of driver sleepiness in field studies. Two measures of ORS were used: (1) one for behavioural signs based on facial expression, body gestures and body movements labelled B-ORS, and (2) one based on driving performance e.g. if swerving and other indicators of impaired driving occurs, labelled D-ORS. A limited number of observers sitting in the back of an experimental vehicle on a motorway about 2 hours repeatedly 3 times per day (before lunch, after lunch, at night) observed 24 participant’s sleepiness level with help of the two observer scales. At the same time the participant reported subjective sleepiness (KSS), EOG was recorded (for calculation of blink duration) and several driving measure were taken and synchronized with the reporting. Based on mixed model Anova and correlation analysis the result showed that observer ratings of sleepiness based on drivers’ impaired performance and behavioural signs are sensitive to extend the general pattern of time awake, circadian phase and time of driving. The detailed analysis of the subjective sleepiness and ORS showed weak correspondence on an individual level. Only 16% of the changes in KSS were predicted by the observer. The correlation between the observer ratings based on performance (D-ORS) and behavioural signs (B-ORS) are high (r = .588), and the B-ORS shows a moderately strong association (r = .360) with blink duration. Both ORS measures show an association (r>0.45) with KSS, whereas the association with driving performance is weak. The results show that the ORS-method detects the expected general variations in sleepy driving in field studies, however, sudden changes in driver sleepiness on a detailed level as 5 minutes is usually not detected; this holds true both when taking into account driving behaviour or driver behavioural signs.

## Introduction

The problems of driver sleepiness have gained recognition over the last decade. In parallel an increased number of studies on the characteristics of sleepy driving have been carried out and subsequently reported in the literature [1]. Central to all studies on driver sleepiness is *how to measure sleepiness.* Several approaches to measuring driver sleepiness or, rather, the effects of driver sleepiness have been explored in the literature. These include physiological recordings and their scoring [2], [3], non-obtrusive measures like camera recordings [4] and measures of driving performance [5], [6], [7]. Most of these studies have been carried out in simulators. Also subjective estimations of sleepiness have been used in most studies. This is an easily administrated driver sleepiness measure and several studies have shown that increased self-reported sleepiness are closely related to crash risk in driving simulators [2], [6], [8]. All indicators seem to be relatively sensitive to variations in wakefulness level, although they suffer from specific measurements problems such as large inter-individual differences [6] in the response to sleepiness, but also from a vulnerability to external influences not related to sleepiness. This may be one reason for the difference between the results from simulators versus real driving [9].

A recent trend in studies of sleepy driving is to carry out large-scale naturalistic data collections with instrumented cars [10]. The advantages with this type of field operational tests is the possibility to study to what extent signs of sleepiness contribute to safety-critical incidents [11], [12]. However, this estimation is depending on the possibility to assess sleepiness in a real life context, if this is possible or not is not clear.

In the large-scale field operational studies, quantification of driver sleepiness is based on observer ratings carried out with in-car video recordings [10]. The judgment of sleepiness is normally based on drivers’ facial expression, body movements, postural changes and duration of eyelid closures [13], [14]. This is a technique first described by [15], which had trained observer-raters to evaluate the level of sleepiness of drivers, using video recordings of the drivers’ faces. The result of their study showed adequate test-retest reliability, inter-rater reliability, and intra-rater reliability. The observer ratings were done after a 15second view of the video. However, this work also used video recordings from a driving simulator study with monochrome low-light level images, which probably ensure better quality than is feasible in a large scale naturalistic driving setting. Furthermore the study did not indicate the extent to which the observers rated the “true drowsiness level.

In some situations the experimenter has to make instantaneous ratings – i.e. in the car during the experiment - of the driver’s sleepiness level based on observations made in the vehicle. For example, when severe sleepiness is reached, the experimenter needs to judge if the test drive has to be prematurely terminated due to safety risks (i.e. avoid a crash). The judgment of other individuals’ sleepiness levels is also critical in other real-life person-to-person situations. For example, checking a co-worker’s level of wakefulness is a critical component in fatigue risk safety management in aviation, in order to judge the crew’s fitness-for-duty status. In clinical settings, the medical doctor’s ability to accurately judge the patient’s level of sleepiness may affect clinical diagnosis and choice of treatment [16].

One may assume that observer ratings that are made in the car are more difficult to carry out compared to the video-based approach that has been used in the large naturalistic field studies. Thus, the observer has to integrate various cues related to the body movements, facial expression, eye closure, and driving performance, which all demand sustained attention. Darkness during night time may also impair the accuracy of the ratings. It is also important that the method is reliable and consistent across different observers. We are not aware of any study that has examined observer rated sleepiness in the context described above. However, in an experimental lab study untrained observers, using photographs of the test person’s face presented during 6 seconds, managed to identify sleep deprived individuals as more tired than when the individuals had a normal night of sleep [16]. This finding suggests that humans are sensitive to facial cues and supports that instantaneous ratings of driver sleepiness is a potentially interesting method for quantification of the wakefulness level.

Thus, the aim of the present study was to explore if observer rated sleepiness (ORS) is a feasible method for quantification of driver sleepiness in field studies. Two measures of ORS were used: (1) one for behavioural signs based on facial expression, body gestures and body movements labelled B-ORS, and (2) one based on driving performance e.g. if swerving and other indicators of impaired driving occurs, labelled D-ORS. This pilot study seeks to explore the following questions:

1. Are D-ORS and B-ORS sensitive to different levels of driver sleepiness due to extended time awake, night driving and time on task?
2. How does D-ORS correlate with B-ORS?
3. How do the D-ORS and B-ORS measures correlate with other, established, measures of driver sleepiness?

## Methods

### Participants

In total 24 participants with an equal distribution for gender was recruited for the study. The participants selected were in the age range of 25–65 years old (the average age of the recruited test subjects was 35.4 years) and had a driving experience of more than 5000 km during the year previous to the study. They were recruited with the help of the VTI register of volunteers. The exclusion criteria were need to wear glasses, pregnancy shift working, travelling across at least three time zones during the past two weeks; sleep or health problems, and use of drugs. The participants filled out sleep/wake diaries during the three days before the start of in the study. When filling out the sleep/wake diaries the participants also practiced at giving subjective sleepiness estimations using the Karolinska Sleepiness Scale [17] (KSS). When arriving at the laboratory (and before the first driving session) the participants filled out a background questionnaire and signed an agreement of confidentiality. They also signed an informed consent form. The compensation received for participating in the study was approximately 330 Euro.

### Procedure

The study was carried out during the spring of 2011 on the motorway “E4” from Linköping to Jönköping (Sweden) and back. Each of the 24 participants carried out three driving sessions. Each day two drivers participated and their sessions overlapped. The first participants drove the first session between 09:15 and 11:40, the second session at 15:30 to 17:55 and the last session between 23:30 and 01:55. The second participant drove the first session between 12:30 and 14:55, the second between 18:30 and 20:55 and the final one between 02:30 and 04:55. The drivers were served traditional Swedish warm food at lunch (between 11h and 12h) and dinner around 18h. While driving, the participants reported subjective sleepiness using the Karolinska Sleep Scale every five minutes. The test leader in the front seat instructed the participant when to do this by saying “KSS”. The participant reported verbally value corresponding to an average the last five minutes. The test leader observer in the back seat was responsible for the functioning of the equipment and also for the rating of the both ORS ratings once each five minutes, but one minute before the test subject reported the KSS.

In between the driving sessions the participants stayed at the laboratory of VTI, Linköping Sweden. The experimental car was a Saab 9–3 Aero (model year 2008) which was equipped with double command at the front right passenger seat. This seat was used by the test leader/safety monitor. In addition to the test leader observer in the back seat was responsible for the functioning of the equipment and also for the rating of the both ORS ratings once each five minutes, but one minute before the test subject reported the KSS.

This experiment was based on the governmental approval (N2007/5326/TR) and ethical approval by the Regional Ethical Committee in Linköping, Sweden (EPN:142-07; EPN 142-07 T34-09). The participants received both written and verbal information and instructions beforehand and at arrival to the laboratory, it was underlined that they had the right to stop whenever they wanted without explanation; they signed a written informed consent before the experiments started. This was in line with the Helsinki declaration and accepted by the ethical committee.

### Observers

Six researchers/observers participated in the experiment and they were allocated to the driving sessions as described in Table 1. Due to various constraints it was not possible to balance the observers over the driving sessions in this study.

**Table 1:** Observers (O) allocation over the three driving sessions.

Observer	O1	O2	O3	O4	O5	O6
Session 1	24	–	–	–	–	–
Session 2	–	19	4	1	–	–
Session 3	–	4	4	–	10	3

### Measures

Several measures of driver sleepiness were sampled throughout the experiment, including driving behaviour based measures, physiological signals, subjective estimations of sleepiness given by the participants (i.e. the drivers) and the ORS estimated by the observer sitting in the back seat during the drive. The six signals and measures of interest to the questions considered here are: the lateral position (LP) the standard deviation of the lateral position (SDLP); average blink duration (BLINKDUR); subjective estimations of sleepiness (KSS); observer rated sleepiness with regard to behavioural signs of sleepiness (B-ORS) and, finally, observer rated sleepiness with regard to driving behaviour (D-ORS).

### Lateral position and standard deviation of lateral position

The lateral position was measured using a commercial lane tracker (http://www.mobileye.com/products), which sampled the lateral position of the vehicle at 40 Hz. SDLP was defined as the standard deviation of the distance to the (closest) left lane marking. Segments including a lane change were excluded from the dataset before calculating SDLP.

### Blink duration

The blink duration was measured using electrooculogram (EOG). A Vitaport system was used to record the EOG and the electrodes were of the disposable, self-adhesive, type. Four electrodes were used to record the EOG; one vertical channel (right) and one horizontal channel. The EOG was DC-recorded with a sampling frequency of 512 Hz. The EOG data were processed for analysis of blink duration using a MATLAB program, which determines blink duration based on the mid-slope (50–50) of the triangular EOG pattern that characterizes a blink [18].

### KSS

The Karolinska Sleep Scale (KSS) where used to capture the participants experience of sleepiness. The scale is nine graded and goes from: 1  =  very alert to 9  =  very sleepy, great effort to keep alert, fighting sleep) [17]. In one of the analyses the KSS was divided into three groups where KSS 1–5 correspond to alert, KSS 6–7 correspond to first signs of sleepiness and KSS 8–9 correspond to severe sleepiness. This has been proven to be useful in earlier studies [19].

### ORS

**Figure 1:**
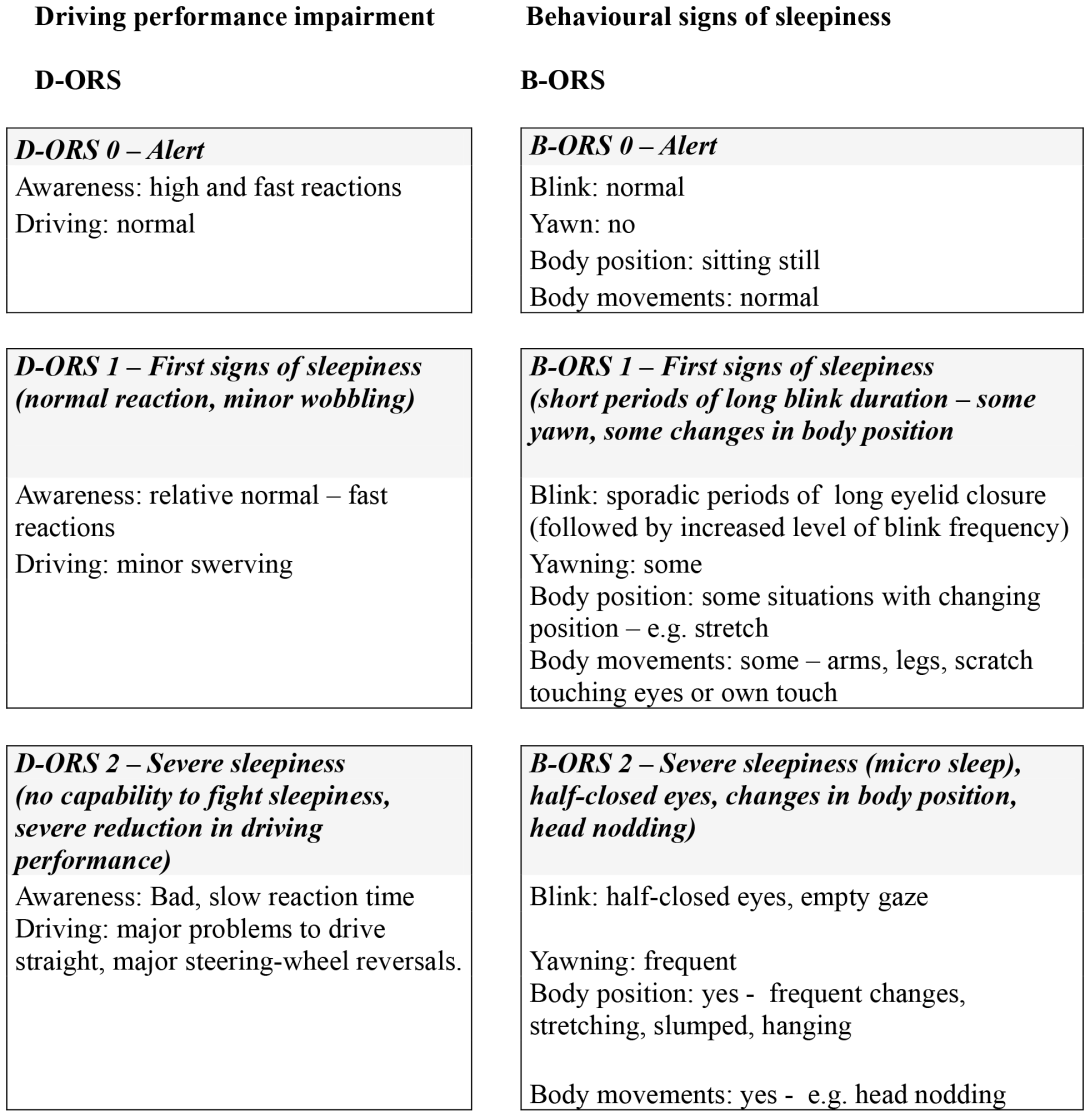
Description of the ORS instruments. Footnote: B-ORS = behavioural sleepiness, D-ORS = driver impaired sleepiness.

The development of the ORS measurement was based on the technique described in the paper by [15]. The objective with the used scale was to describe behaviours that characterize sleepy driving and was inspired by the study of [14]. The observed behaviours could be categorized into the following basic categories: eye-related behaviours (e.g. long eye closure, slow blink rate), facial movements (e.g. yawning), body movements (e.g. stretching, moving trunk forwards backwards) and risky driving behaviour (e.g. driving on rumble strip, swerving, large steering wheel corrections). The categories were grouped into two ORS scales and the observer was to give an estimate on each scale. As a support the observer had a video screen of the drivers face to look at. The two scales were one for driving impairment (D-ORS) and one for the driver's behavioural sign of sleepiness e.g. blink behaviour and body position (B-ORS). There were three levels for each scale: 0 = ”Alert”; 1 = ”First signs of sleepiness” and 2 = ”Severe sleepiness”. The scale developed by Wierwille and Ellsworth [15] used five response categories, however, in order to reduce error variance it was decided to decrease the number of response levels to three. The guidelines for the two ORS scales are presented in Figure 1. The instruction to the observer was that there was no need for major changes in all behaviour within one ORS category to justify a change in an ORS level.

### Statistical analysis

It has been shown that most indicators of driver sleepiness should be computed for intervals of 60 seconds duration or longer in order to give fair indications of a driver's level of sleepiness [20]. Based on these findings, in combination with a wish to obtain as many valid indicator measures as possible, the data were divided into intervals of one minute and all indicators (other than KSS and ORS) were computed for all the one-minute intervals. To make comparisons between KSS and ORS, which were sampled every five minutes, the indicators were averaged over the *valid* one-minute intervals out of the five one-minute intervals corresponding to each five-minute interval defined by the KSS estimations. A one-minute interval was deemed valid if the following criteria were fulfilled: (1) The speed limit was 110 km/h or higher, (2) the average speed over the interval was at least 90 km/h, (3) the lane tracker had high confidence and (4) no driving with lane changes. All data between minutes 45 and 60 have been excluded since the drivers reached the turning point on the motorway and drove back to Linköping. In the end of the drive during the night drive only 7 out of 24 participants manage to finalize the driving session and this is the reason for the drop in data for the night time session.

In order to see if D-ORS and B-ORS are sensitive to the study design parameters, the three driving sessions ((1) before lunch, (2) after lunch and (3) night) and time on task (time driven), a full factorial mixed model Anova with subject as random factors, was used. To see how D-ORS and B-ORS correlate with KSS but also with other measures of sleepiness the correlations between the considered measures were computed and the significance was tested with a non-parametric approach (Kendals TauB). To obtain a three-level KSS-scale to compare with the ORS the KSS was divided into three levels conceptually similar to the three levels of the ORS. The relation between the KSS and the ORS was the following: KSS 1–5 correspond to ORS 0, KSS 6–7 correspond to ORS 1 and KSS 8–9 correspond to ORS 2. Differences between the observers’ B-ORS and D-ORS estimations were analysed with consideration to the drivers’ KSS estimations. However, no significant difference between the two main observers working during session 2 or between the four main observers working during session 3 was seen.

## Results

### The effect of night driving on performance and sleepiness

**Figure 2:**
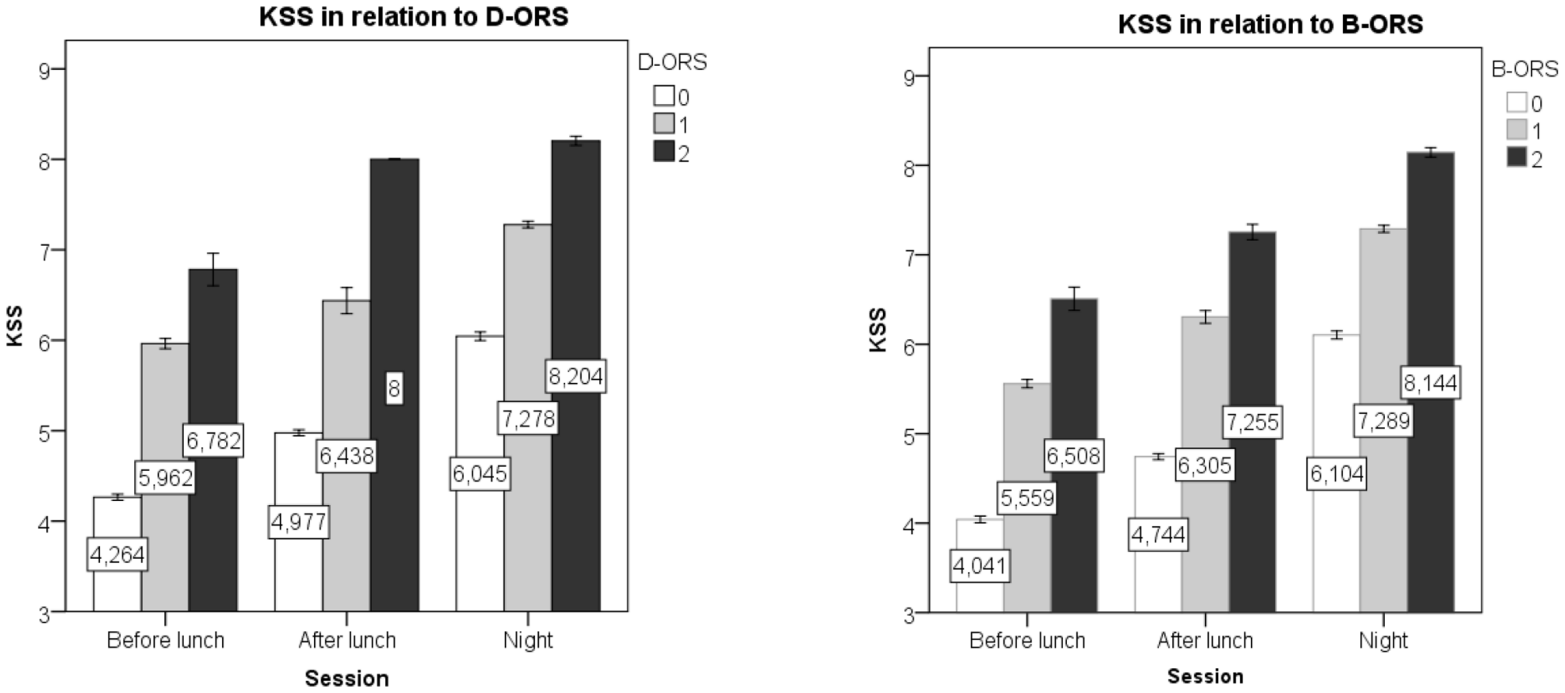
KSS in relation to B-ORS and D-ORS. Footnote: The lines are separated for the design parameter driving session (before lunch, after lunch and night). Error bars represent SE mean.

The KSS were in average 4.7 for B-ORS0; 6.2 for B-ORS1 and 7.5 for B-ORS2. The difference a cross the three levels was significant (F = 200.0; p<0.01), see Figure 2. In addition there was a significant interaction for Session*B-ORS (F = 5.3;p<0.01). The KSS was in average 4.8 for D-ORS0; 6.6 for D-ORS1 and 7.9 for D-ORS2 and also here the difference were significant (F = 61.6;p<0.01) and with an interaction between D-ORS and Session (F = 24.3;p<0.01).

**Figure 3:**
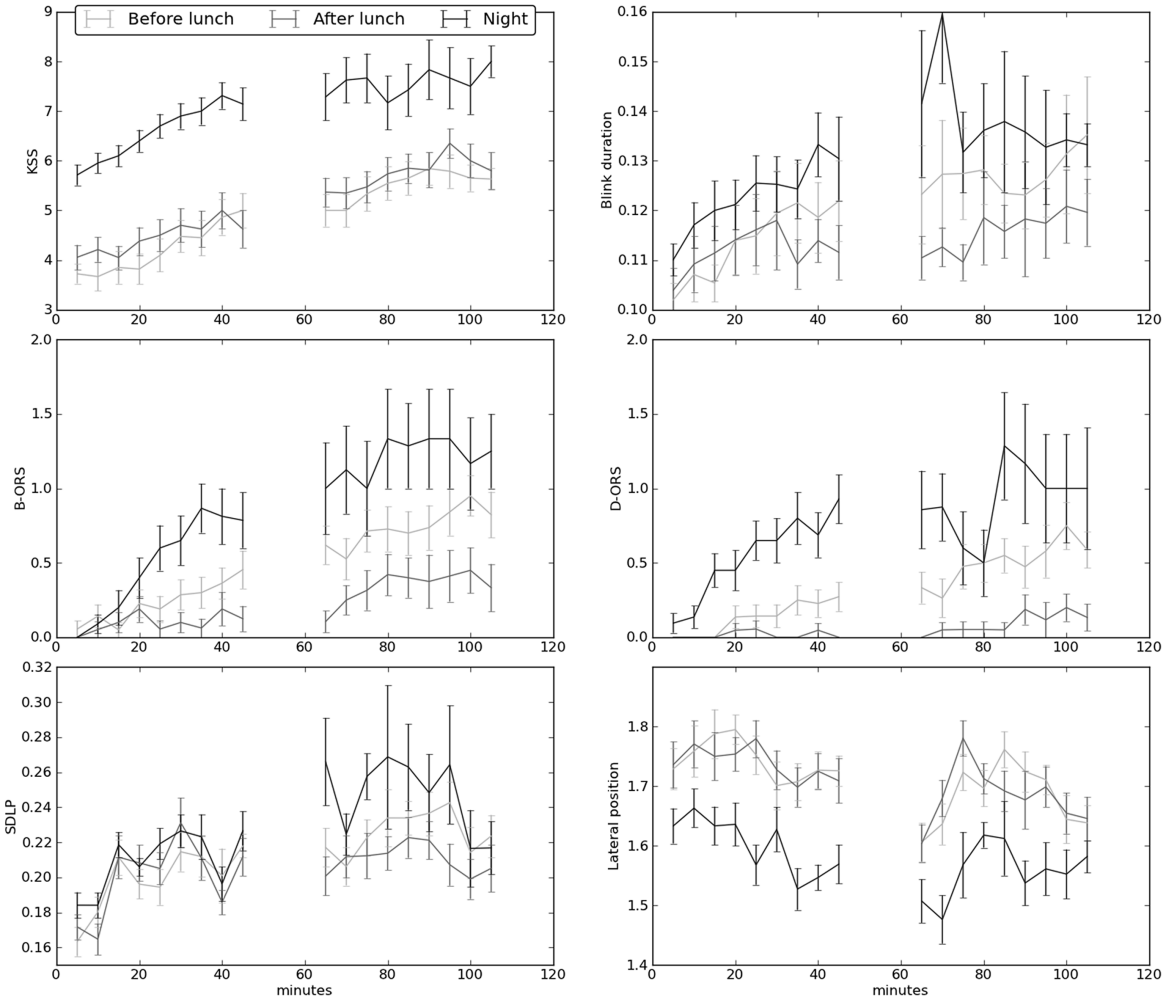
Drivers’ sleepiness and driving performance in relation to design parameters. Footnote: Driving session (before lunch, after lunch and night) and time on task (Minute 0–45 and 60–105). Error bars represent SE mean. SDLP = standard deviation of lateral position. KSS = Karolinska Sleepiness Scale.

Observer rated sleepiness (D-ORS and B-ORS) increased significantly during night time driving and with time on task (minutes driven). So did also subjective estimations of sleepiness (KSS), blink duration (BLINKDUR), lateral position (LP) and the standard deviation of lateral position (SDLP). There was a significant interaction between session and time on task for all measures. In addition the differences between participants were significant for all measures and their interactions, except for the main effect for D-ORS. The highest F-values were seen for KSS, D-ORS and B-ORS, see Figure 3 and Table 2.

**Table 2:** Mixed model ANOVA.

	Session (F;p<)	Minutes (F;p<)	Minutes*Session (F;p<)	Particip. *(random)* (Wald Z;p<)	Particip. *Session (Wald Z;p<)	Particip. *Minutes (Wald Z;p<)
KSS	**68.7 (0.01)**	**71.2 (0.01)**	**13.0 (0.01)**	**2.38 (0.01)**	**12.78 (0.01)**	**4.68 (0.01)**
BLINKDUR	**38.5 (0.01)**	**8.10 (0.01)**	**3.48 (0.01)**	**3.23 (0.01)**	**11.57 (0.01)**	**4.38 (0.01)**
LP	**35.8 (0.01)**	**13.3 (0.01)**	**2.6 (0.01)**	**2.88 (0.01)**	**7.70 (0.01)**	**4.02 (0.01)**
SDLP	**34.8 (0.01)**	**3.4 (0.01)**	**1.7 (0.01)**	**2.11 (0.01)**	**3.18 (0.01)**	**2.69 (0.01)**
B-ORS	**48.8 (0.01)**	**32.8 (0.01)**	**33.4 (0.01)**	**2.43 (0.01)**	**12.09 (0.01)**	**4.54 (0.01)**
D-ORS	**60.99 (0.01)**	**26.50 (0.01)**	**42.74 (0.01)**	1.83 (0.07)	**12.09 (0.01)**	**4.54 (0.01)**

Footnote: KSS = Karolinska Sleepiness Scale, LP = lateral position, SDLP = standard deviation of lateral position, B-ORS = behavioural signs of observer rated sleepiness, D-ORS = driving impairment related observer rated sleepiness, Session (df = 2):before lunch, after lunch, night) and Time (df = 17) (minute 1–45 and 65 to 105). F and p-values. Significant values in bold.

### Correlation between B-ORS and D-ORS and other measures of sleepiness

B-ORS and D-ORS was highly, but not perfectly, correlated (r = 0.588), see Table 3. Both B-ORS and D-ORS were correlated to KSS, BLINKDUR and to LP. D-ORS was also correlated to SDLP. KSS had a significant correlation to BLINKDUR, LP and SDLP. BLINKDUR was not correlated to either LP or SDLP, on the other hand the highest correlation was seen for BLINKDUR and B-ORS. Even though the correlations between variables were significant only the correlation between B-ORS and D-ORS; KSS and B-ORS; KSS and D-ORS; BLINKDUR and B-ORS; BLINKDUR and D-ORS; and finally BLINKDUR and KSS was greater than r = 0.24.

**Table 3:** Correlation; significant (p<0.01) correlations are in bold.

	B-ORS	D-ORS	KSS	BLINKDUR	LP	SDLP
B-ORS	1.000	**0.588**	**0.437**	**0.360**	–**0.077**	0.015
D-ORS		1.000	**0.449**	**0.271**	–**0.108**	–**0.044**
KSS			1.000	**0.237**	–**0.137**	–**0.077**
BLINKDUR				1.000	–0.003	0.006
LP					1.000	–0.006
SDLP						1.000

### Analysis of changes in KSS

In total there were 921 observations for B-ORS. From an overall perspective, comparing the results for KSS (reported once every 5 minutes with the B-ORS (reported 1 minute before the subject reported the KSS level) 73% (677) of the classifications were consistent with each other. When KSS was grouped into three categories (KSS 1–5, KSS 6–7 and KSS 8–9) to represent levels of low, intermediate and high self-rated sleepiness [17] 162 situations were obtained in which the level of KSS increased or decreased. Table 4 describe the KSS category and the corresponding changes (if any) in B-ORS measures. There were 73 decreases in KSS level and 31(42%) were predicted by the observer in the B-ORS rating. There were 89 increases in the KSS and for 15 (17%) of them the observer rated an increase in B-ORS. In total, for the 162 changes in KSS 46 changes (28%) were preceded by a correct change in B-ORS level.

**Table 4:** Cross tabulation of changes in KSS grouped and the corresponding changes in ORS.

	All sessions					
	dB-ORS			dD-ORS		
**dKSS**	–**1**	**0**	**1**	–**1**	**0**	**1**
–**1**	31	35	7	31	39	3
**0**	51	631	77	38	656	65
**1**	5	69	15	1	76	12

Footnote: (dB-ORS = difference in B-ORS; dKSS = difference in KSS).

**Figure 4:**
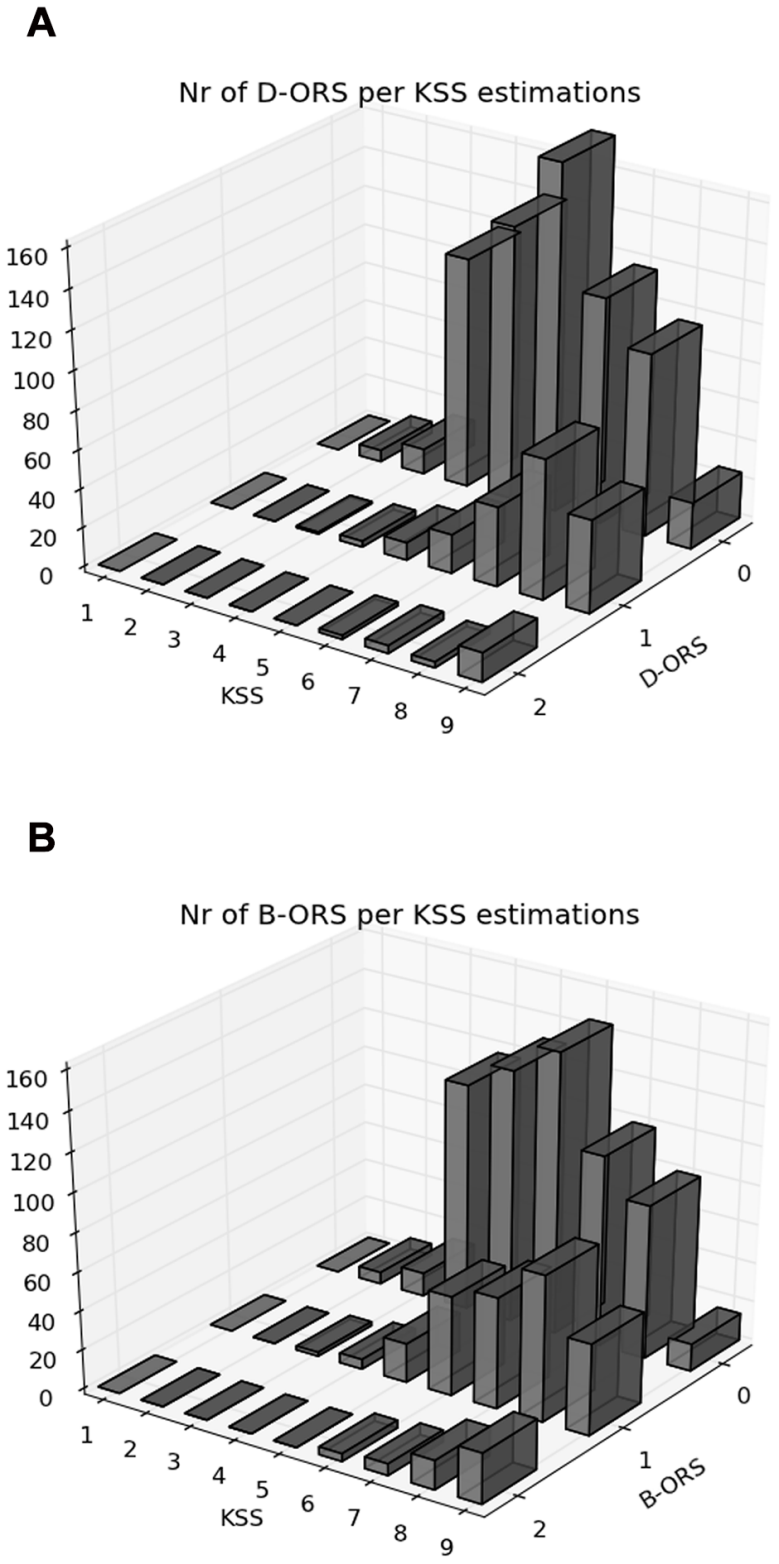
The relation between B-ORS/D-ORS and KSS scoring for each separate level of KSS.

For the D-ORS the results were almost the same with a total hit rate of 76% (699/921); a hit rate of an increase in KSS of 13% (12/89) and 42% (31/73) for the decrease in KSS level. In total, for the 162 changes in KSS 43 changes (27%) were preceded by a correct change in D-ORS level. No systematic difference in these results could be seen between the three driving sessions. A detailed description of the relation between B-ORS/D-ORS measures and KSS is presented in figure 2 and in figure 4.

To control if the observer was influenced by the participant’s reported KSS four minutes before the observer reported, the correlation was computed. The lagged correlation between a given KSS value and the B-ORS value 4 minutes after was 0.21. The corresponding value for D-ORS was 0.21.

## Discussion

The results showed that ORS based on either behaviour or impaired driving performance was sensitive to well-known manipulations of sleepiness such as night driving, extended time awake and increased time of driving. Furthermore, the ORS measures showed correlations with both the drivers’ own ratings of sleepiness and with an objective indicator of driver sleepiness; blink duration. This suggests that observers in the car can detect variations in driver sleepiness when the general patterns change. In addition, the results show that it may be difficult to foresee a change in KSS beforehand using a five minute interval. Thus, observer ratings have not been proven to be better indicators of driver sleepiness or driving impairment due to sleepiness than objective measure as BLINKDUR, LP or SDLP.

Interestingly, the KSS values within the B-ORS values increased systematically across the three sessions. For B-ORS 0 mean values ranged between 4.0 and 6.0, for a value of B-ORS 1 KSS ranged from 5.5 to 7.1, and for B-ORS value 2 they ranged between 6.4 and 8.1. Similar results were seen for D-ORS. Thus, the self-rated sleepiness increased with sessions for a given ORS value. This does not seem to be a problem since each ORS-level should cover a certain range in self-rated or other indices in sleepiness.

The ORS measures showed the expected time of day pattern. During daytime driving before lunch, when the highest level of alertness was expected, mean ORS was close to 0. A marginal time of driving increase was observed and ORS almost reached 0.5 towards the end of the early daytime drive. The ORS levels were somewhat higher during daytime driving after lunch, which might be related to driving during the afternoon dip. The highest ORS ratings were observed during night driving. Although, both ORS measures started at a low level in the beginning of the night drive, the mean level almost reached 1 after 30 minutes of driving. During the second half of the night drive the mean ORS levels reached almost 1.5 towards the end of the 1.5-hour driving session. This corresponds with KSS ratings ≥7, which is the level where physiological signs of sleepiness appear such as increased blink duration [17].

One aim of the study was to explore if ORS based on driving impairment differed from ORS based on driver behaviour, such as facial cues, body movements and body gestures. The mean levels and the temporal pattern for the sessions for both ORS measures were very similar. The similarity was also demonstrated in the high correlation coefficient (r = 0.588) between the two measures. Thus, driving behaviour seemed to be equally sensitive as facial cues and body movements when the drivers’ level of sleepiness was quantified. Both ORS measures showed correlations with KSS, whereas ORS-B had slightly higher correlation with blink duration (r = 0.36) than D-ORS (r = 0.27). This suggests that eye-related cues were an important input source for B-ORS. The assumption that D-ORS should be associated with objective driving performance was not supported by the data. Although the correlation was significant, the magnitude was very low (r<–0.04) and the direction was unexpected (e.g. higher sleepiness was associated with reduced standard deviation of the lateral position). However, it is also notable that none of the other sleepiness indicators showed any substantial correlations with driving performance indicators. Our previous field studies suggest that measures of driving performance are not feasible indicators of driver sleepiness due to large individual differences and measurement error [21], although field studies from other groups have shown that lane crossings are sensitive to increased levels of sleepiness [7]. In sleepiness studies on real roads there are confounding factors caused by changes in the context like sections with wider lanes, different road markings, pedestrians on shoulder, on-coming vehicle etc. This may explain the problems to identify changes in driving performance under real driving, problems that we do not have in the same way in data from driving simulators.

Although the classification between B-ORS and re-scored KSS into three groups (1–5: alert, 6–7 sleepy, 8–9 very sleepy) was correct in 73% of the events (76% for D-ORS) the results indicate difficulties for an observer to detect sudden changes in driver sleepiness. Thus, when the driver reported increased KSS, ORS was in most cases unaffected. The lack of ability to determine whether a driver has developed an increased sleepiness in a given time interval of 5 minutes is not in line with previous research [12] and the reason for the deviation is not known and further studies are of interest. One obvious difference between this study and other similar studies is that the observer is a passenger in the in the car and bases the observation on real time experience, taking into account also other cues that may be relevant but not included in the rating scale. Probably, it may also be that a sudden increase in KSS does not result in a change in facial cues, body movements or driving behaviour. Furthermore, self-rated sleepiness (KSS) may not be the perfect reference for driver sleepiness. Although KSS in previous driving studies has been associated with relatively low between-individual variance, at least compared to objective indicators of driver sleepiness [8], one may assume that some individuals can’t accurately assess their level of sleepiness. It may also be that ORS measures the consequences of sleepiness rather than sleepiness per se. Roge et al [14] suggested that body movements and to some extent also eye movements reflect fighting sleepiness. Thus, it cannot be ruled out that an increase in self-rated sleepiness may not always result in a change in facial cues, body movements or impaired driving performance.

In line with this B-ORS should be more sensitive to reflect sleepiness, but also more related to physiological sleepiness signs. Also this is supported by the fact that B-ORS and BLINKDUR show the highest correlations. On the other hand it might be that the D-ORS is more sensitive to the effect of sleepiness that may be observed in terms of measures of driving performance. However, a lower correlation with BLINKDUR was seen, but not a higher correlation with driving behaviour measures. If the driver is fighting sleepiness the B-ORS score will probably increase, whereas the other indicators might decrease. Thus, one should perhaps not expect a strong correlation between B-ORS and other measures of driver sleepiness.

This study suffers from several limitations, of which one of the major ones is that it may be that the observer ratings are biased by the KSS reported 4 minutes before. However, this does not seem to be the case since the (lagged) correlation was low (0.21) between both B-ORS and D-ORS and the preceding KSS. However, future studies are recommended in which the participant’s scores are blinded to the observer. The driver may also be biased by the fact of the time of day. There is a risk that they overestimate sleepiness night time just by the fact it is night time. A second limitation is the number of observers. During evening and night sessions there were only few observers, and even though the results did not show any difference between them there is a need to be extra cautious. It could also be discussed if the total number of observation and the distribution is enough. Future studies about the effect of individual and intra individual changes among observers are recommended. The comparison between KSS and B-ORS/D-ORS is depending not only on the correctness in B-ORS/D-ORS but also on KSS as a true value. A limitation here is that this is not by default true, which has already been discussed. The correlation between the KSS and BLINKDUR is less than between KSS and B-ORS/D-ORS, but also less than B-ORS and BLINKDUR. It may indicate that the drivers underestimate their sleepiness. The relation between drivers’ own perception of sleepiness and objective indicators of driver sleepiness needs further studies. It could also be discussed if it is correct to use a parametric test instead of a non-parametric test for the analysis of the D-ORS and B-ORS. In this case it was important to also look into the issue of interactions with other indicators that we know are normal distributed and therefore we used an Anova. Finally one limitation is about the possibility to do estimation on two dimensions; driving and driver related. It could be discussed if the observers really can remember all actions with a time frame of 5 minutes, and also to separate them. The observer is put in a difficult situation and it is not known if two different judgements are possible to do with the same accuracy.

## Conclusion

Observer ratings of sleepiness based on drivers’ impaired performance and behavioural signs show sensitivity to extended time awake and night driving. The changes in B-ORS and D-ORS follow the pattern from other indicators of sleepiness like self-reported sleepiness (KSS). The detailed analysis of the changes in KSS and B-ORS or D-ORS showed major difficulties on an individual level were only 16% of the changes in KSS were predicted by the observer. The correlation between the observer ratings based on performance (D-ORS) and behavioural signs (B-ORS) are high, and the B-ORS shows a stronger association with blink duration than D-ORS. Both ORS measures show a strong association with KSS. The results indicate difficulties for an observer to rate changes in driver sleepiness on a detailed level as 5 minutes; this holds true both when taking into account driving behaviour or driver behavioural signs.
